# Application of Artificial Neural Network Based on Traditional Detection and GC-MS in Prediction of Free Radicals in Thermal Oxidation of Vegetable Oil

**DOI:** 10.3390/molecules26216717

**Published:** 2021-11-06

**Authors:** Shengquan Huang, Ying Liu, Xuyuan Sun, Jinwei Li

**Affiliations:** 1 Nuspower Greatsun (Guangdong) Biotechnology Co., Ltd., Guangzhou 510931, China; Huangfeihong0220@sina.com; 2 School of Food Science and Technology, Jiangnan University, Wuxi 214122, China; liuying19891120@sina.com (Y.L.); xysun@jiangnan.edu.cn (X.S.)

**Keywords:** free radical, electron paramagnetic resonance, volatile, lipid oxidation, artificial neural network (ANN)

## Abstract

In this study, electron paramagnetic resonance (EPR) and gas chromatography-mass spectrometry (GC-MS) techniques were applied to reveal the variation of lipid free radicals and oxidized volatile products of four oils in the thermal process. The EPR results showed the signal intensities of linseed oil (LO) were the highest, followed by sunflower oil (SO), rapeseed oil (RO), and palm oil (PO). Moreover, the signal intensities of the four oils increased with heating time. GC-MS results showed that (*E*)-2-decenal, (*E*,*E*)-2,4-decadienal, and 2-undecenal were the main volatile compounds of oxidized oil. Besides, the oxidized PO and LO contained the highest and lowest contents of volatiles, respectively. According to the oil characteristics, an artificial neural network (ANN) intelligent evaluation model of free radicals was established. The coefficients of determination (R2) of ANN models were more than 0.97, and the difference between the true and predicted values was small, which indicated that oil profiles combined with chemometrics can accurately predict the free radical of thermal oxidized oil.

## 1. Introduction

Vegetable oils, such as palm oil (PO), rapeseed oil (RO), sunflower oil (SO), and linseed oil (LO), are essential components in the human diet [1]. However, the oxidation and hydrolysis of lipids occurs in high-temperature cooking and frying, causing loss of nutrition, deterioration of quality, unpleasant flavors, and the formation of toxic compounds [2]. Extensive studies on lipid autoxidation have been performed in recent decades focusing on the analysis of traditional chemical indexes and oxidation hazard products [3,4,5]. However, the thermal oxidation of lipids at high temperatures is a more complex process than autoxidation as high temperature accelerates the reactions between fatty acids. Additionally, some secondary products that are different from the typical oxidation products are formed under oxygen-deficient conditions. Furthermore, the high energy input triggers the reaction easily, while the propagation and termination of free radicals at high temperatures make the process complex [6].

Among the oxidative products, volatile products are generated due to the breaking of fatty acid chains, resulting in the overall flavor of the oil. The flavor constitutes hundreds of volatile compounds, particularly in RO [7,8]. The flavor and volatile products vary widely depending on oil type, processing technique, and oil quality. Krist et al. [9] found that the main volatiles of linseed oil were trans- 2-butenal and acetic acid, while trans-2-pentenal, α-pinene, trans-2-heptenal, trans-3-octen-2-one, and trans,trans-2,4-heptadienal were the dominant volatile compounds. Considering the flavor discrimination of edible oil with different extractions, Dun et al. [10] compared hot-pressed and cold-pressed peanut oil and found that the volatiles in hot-pressed peanut oil presented a fresh, fatty, nutty, and baking flavor, while volatiles obtained from the cold-pressed peanut oil showed a fresh and nutty flavor. Upon heating at high temperatures, the pleasant flavor is generated by the rapid cleavage of fatty acid chains [11]. However, the flavor of oil deteriorates during lipid oxidation due to the pyrolysis and polymerization of the fatty acid chains. Therefore, these volatile compounds are the direct evaluation indicators of lipid oxidation.

Several techniques have been used to investigate the lipid oxidation process, including nuclear magnetic resonance (NMR) [12,13], near infrared spectroscopy (NIRS) [14], gas chromatography-mass spectrometry (GC-MS) [15], liquid chromatography tandem mass spectrometry (LC-MS/MS) [16], and fluorescence spectroscopy [17]. There has been a growing interest to invent novel methods, such as digital image colorimetric analysis, supramolecular chemistry, and other sensitive techniques for the analysis of minor oxidation products in lipid oxidation [18,19,20,21]. The mechanism of lipid oxidation is the chain reaction of free radicals. However, most of the methods discussed above are based on the changes in the fatty acid chains, while only a few are applicable to the analysis of lipid free radicals. As the oxidation proceeds, fatty acids are degraded into short chains, including peroxyl, lipid, alkoxyl, and hydroperoxyl radicals. Free radicals in lipids are reactive and unsteady. Recently, electron paramagnetic resonance (EPR) spin trapping has been widely applied to determine and identify the radicals in food and lipid [22,23]. This technique allows for the analysis of the changes in free radicals in a complex reaction system with the advantages of accuracy and sensitivity. However, the EPR technique for detection of lipid free radicals involves the use of trapping agents, such as 5,5-dimethyl-1-pyrroline N-oxide (DMPO) and α-phenyl-N-tert-butylnitrone (PBN), which are unstable and prone to degrade in light and humid conditions. Besides, the EPR detection is performed at high temperature and is time-consuming [23].

An artificial neural network (ANN) was applied in food to assess aspects of a kinetic model, physicochemical properties, and to facilitate quality analysis and intelligent control design [24]. Generally speaking, an ANN is used to predict product indicators with the inputs of parameters [25]. Husna and Purgon (2016) used a BP-ANN model to predict the moisture content of durian slice with the mass, temperature, thickness, and drying time as inputs [26]. Sun et al. (2019) established the ANN of flavor changes in ginger during microwave vacuum drying based on LF-NMR parameters [27]. Sun et al. (2019) applied ANN to monitor water states of typical fruits and vegetables after drying treatments [28]. Besides, ANN has been successfully applied in cell biology and diseases. For example, Le et al. established a reliable method for biologists to use SNARE identification by ANN, which provided a basis for applying a fastText word embedding model into bioinformatics [29]. Do et al. found that an ANN model achieved excellent performance in predicting S-sulfenylation sites compared to other well-known tools on a benchmark dataset [30].

The free radicals formed by the degradation and oxidation of fatty acids during the thermal process should be investigated as these could reflect the degree of thermal oxidation of lipids and oil. In this study, the traditional chemical indexes involving the thermal oxidation of vegetable oils were collected, and the lipid free radicals of oils under different heating temperatures were studied by EPR. At the same time, the flavor change of oils during heating was determined by gas chromatography mass spectrometry (GC-MS). The characteristic volatiles of oils were selected by hierarchical clustering analysis (HCA). The free radical prediction models of vegetable oils during the thermal process were established by ANN aided by chemical properties and GC-MS. This study is expected to provide an intelligent method for the fast and accurate detection of free radicals of vegetable oils during the thermal process.

## 2. Results and Discussion

### 2.1. Free Radical Analysis under Thermal Process

**Figure 1:**
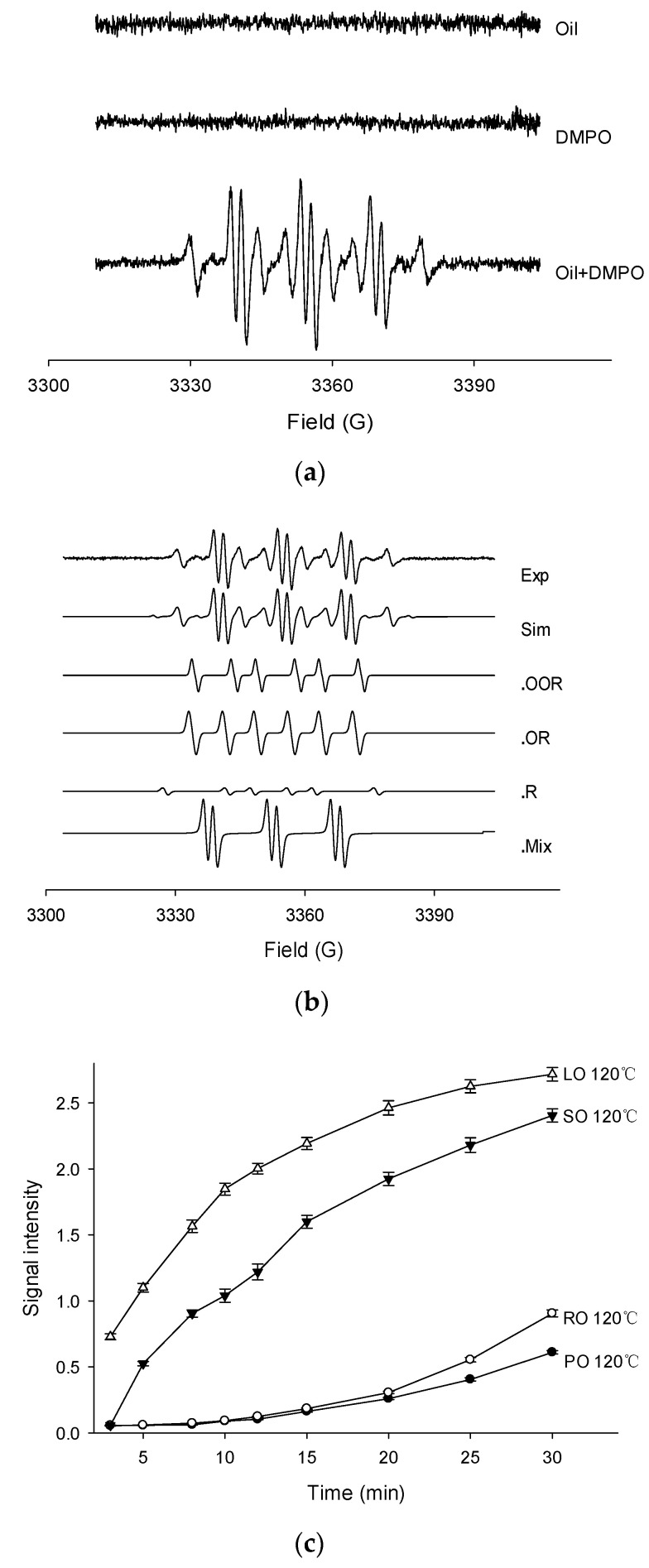
The EPR spectrums of palm oil (**a**), experimental and simulated EPR spectrums of palm oil (**b**) and signal intensities of four oils heated at 120 °C (**c**). (Note: Exp, experimental spectrum. Sim, simulation of the EPR spectrum. •OOR, simulation of the DMPO/•OOR spectrum. •OR, simulation of the DMPO/•OR spectrum. •R, simulation of the DMPO/•R spectrum. •Mix, simulation of the DMPO likely adducts. PO, palm oil. RO, rapeseed oil. SO, sunflower oil. LO, linseed oil).

Thermally oxidized oils were examined for free radical production by the EPR spin trapping technique using DMPO to reveal the variation of radicals in four kinds of oil. Firstly, the EPR spectra of palm oil, DMPO solution (control), and PO in DMPO oxidized at 120 °C are shown in Figure 1a. The negligible EPR signal was found in PO or DMPO solution, while a very intense EPR signal was observed in the combination of PO and DMPO. Similar results were also presented by the other three oils with different heat temperatures. The mechanism of successful detection under experimental condition was that the spin trapping technique can form considerably stable spin adducts which are totally different from high reactive and unstable radicals. Most of the samples showed multiple EPR signals. The most obvious adducts were caused by alkyl radicals, and a typical six peaks and some small peaks could be seen. Taking the example of PO, experimental and simulated EPR spectra of PO detected at 120 °C in the presence of DMPO are shown in Figure 1b. The composite spectrum indicated a mixture of four distinct radical adducts. After simulation and calculation of their respective hyperfine splitting constants (a_N_, a_H_ in Gauss) by computer, the spin adducts were assigned as peroxyl (DMPO/•OOR; a_N_ = 14.75, $a_{H}^{\beta }$ = 12.86, $a_{H}^{\gamma }$ = 1.50, g value = 2.00732), alkoxyl (DMPO/•OR; a_N_ = 14.45, $a_{H}^{\beta }$ = 9.18, $a_{H}^{\gamma }$ = 1.17, g value = 2.00711), alkyl (DMPO/•R; a_N_ = 14.31, $a_{H}^{\beta }$ = 20.02, g value = 2.00702), and an DMPO-likely radical adducts with a characteristic three-line EPR spectrum (a_N_ = 14.79, $a_{H}^{\beta }$ = 2.00, g value = 2.00725).

The EPR measurements of four fresh oils were carefully performed under the temperature of 120 °C (Figure 1c). The signal intensity of the EPR spectrum, was taken as an important parameter to indicate the amounts of free radicals. The highest signal intensity was observed in the LO sample, followed by SO and RO, while the lowest was found in PO. In general, the signals increased during the entire period. In the first 10 min, the total spin adducts in SO and LO increased rapidly and then slowed down in the last 10 min, while the signals in PO and RO show low initial increased rates. It can be predicted that the signal intensities of LO and SO reached a plateau after 30 min, while the PO and RO signals showed a steady increase. These opposite trends showed the difference in the oxidative stability of the four oils. The magnitude of the signal in each oil sample was mainly attributed to the double bonds of the unsaturated fatty acids, indicating the stability against oxidation. Accordingly, PO was mainly composed of oleic acid (33.56%) and palmitic acid (29.29%), while RO, SO, and LO mainly consisted of oleic acid (46.29%), linoleic acid (51.87%), and linolenic acid (41.02%), respectively. In the study by Symoniuk et al., this feature was known as the oxidizability value, which could be calculated using the fatty acid profile [31]. Based on the EPR signals, the thermal-oxidative stability of the four oils was in the order of PO > RO > SO > LO, corresponding to the initial OSI results in Table 1. For instance, in the absence of the antioxidants, the oxidative stability of PO can only be attributed to the highest amount of saturated fatty acids. Meanwhile, the free radical signals in PO showed a relatively lower increase rate. Therefore, in 30 min of heating, the trends of the signal intensities in the four oils showed significant differences, which can also explain the different degradation behavior of the four heated vegetable oils.

### 2.2. EPR Analysis of Oxidation Behavior

**Figure 2:**
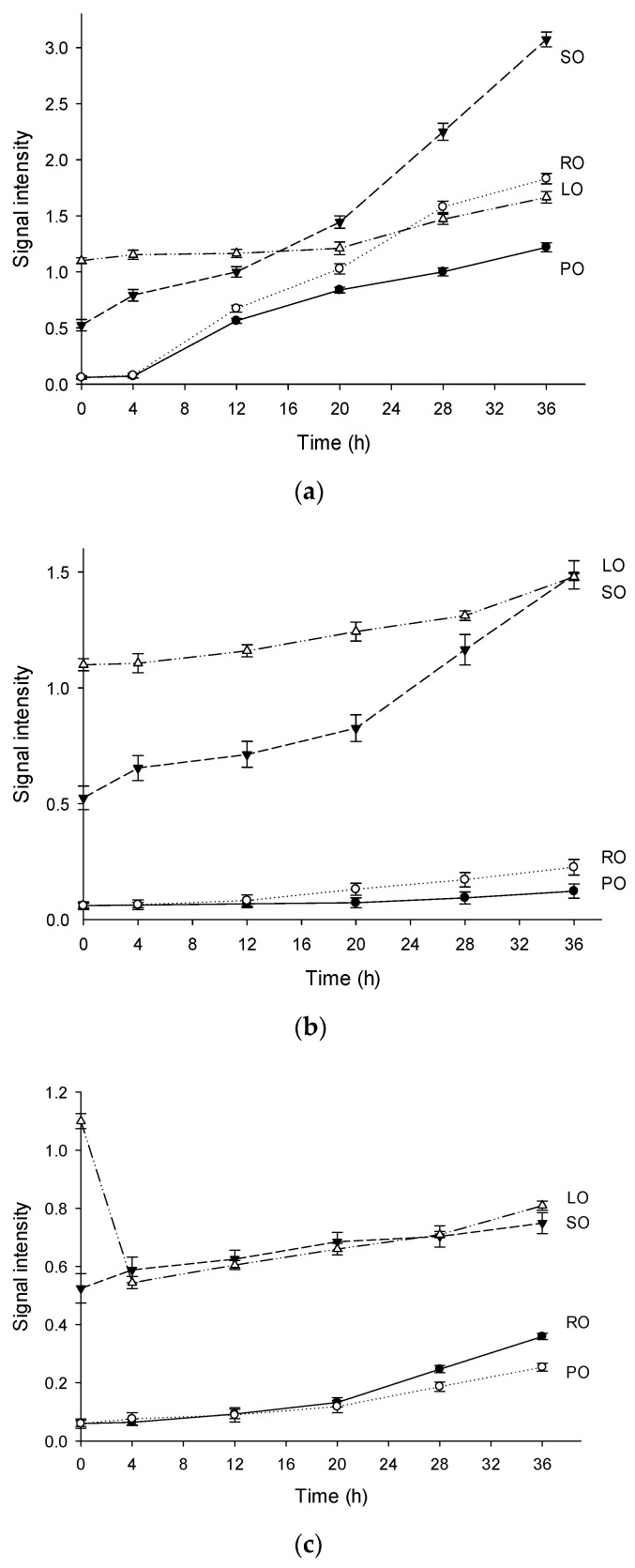
The variation tendencies of signal intensities in four vegetable oils heated at 120 °C (**a**), 150 °C (**b**), 180 °C (**c**). (Note: Exp, experimental spectrum. Sim, simulation of the EPR spectrum. •OOR, simulation of the DMPO/•OOR spectrum. •OR, simulation of the DMPO/•OR spectrum. •R, simulation of the DMPO/•R spectrum. •Mix, simulation of the DMPO likely adducts. PO, palm oil. RO, rapeseed oil. SO, sunflower oil. LO, linseed oil).

Figure 2 also exhibits the signal intensities of the oils heated at 120 °C, 150 °C, and 180 °C. In general, the intensities of the EPR signals in LO and SO were high, while PO and RO exhibited low signal intensities. The signal intensities of the four oils at 0 h in the decreasing order were as follows: LO, SO, RO, and PO. The sequence was in accordance with the PVs (Table 2) of these oils. However, the thermal oxidation of oils is more likely to depend on the fatty acid composition. For instance, the signal intensities of LO were the highest in the first 12 h (Figure 2a,b), owing to the highest linolenic acid content (18:3). Interestingly, the overall signal intensities of LO and SO (high group) at different temperatures were in the following order: 120 °C > 150 °C > 180 °C. This sequence can be a result of the instability of lipid radicals at high temperature. Additionally, the signal intensities of RO and PO (low group) at 150 °C were lower than those at 180 °C. This strongly indicated that the oils with high saturated fatty acid contents could result in relatively low levels of the lipid radicals. It is obvious that oil containing more proportion of saturated fatty acid is more capable of antioxidation. These results were consistent with the previous findings for the kinetic model described by Roman et al. [32].

The signal intensity in LO was the highest in the first 4 h (Figure 2c). As listed in Table 2, linolenic acid was the predominant fatty acid in LO, and was easily transformed into lipid radicals by the free radicals. As previously reported, a high concentration of radicals can increase the destruction rate of the spin adducts [33]. High concentrations of free radicals are unstable at high temperatures, which exacerbate the chain reaction to the termination. This can explain the changes in the signal intensities at 180 °C, where the hydroperoxides degraded rapidly. When heated at 120 °C, the formation rate of the spin adducts was very high in the first 30 min. However, the signal intensities reached a plateau after 30 min, and the fresh radical spin traps added in the samples can react with the stable poly-adducts formed by the carbon chains of the fatty acids [34]. The signals of the spin adduct maintained a constant increase rate after several hours of stabilization (Figure 2c), where the consecutive synthesis and degradation reactions occur simultaneously.

The signal intensity of SO heated at 180 °C was the second highest among four oils and showed a slight increase with the increased heating time, indicating that a balance occurred between the synthesis and degradation reactions of the radical adducts. The saturation of PO was the highest among the four oils, and more energy was required for the thermal cracking of the saturated chains than that for the unsaturated chains. In other words, saturated chains can resist free radical attack and delay the formation of oxidation products. From an electronic perspective, the electron cloud density of the double bond region was low, so it was vulnerable to nucleophilic attack by free radical inducer and free radicals. These unique features of different fatty acids were analyzed by EPR during the radical propagation, which corresponding to the increases of signal intensity in thermal degradation of vegetable oils. Similar results were observed in RO which had a relatively low content of unsaturated fatty acids. The signal intensity of RO and PO was almost the same when heated for 20 h, but after that the signal intensity of RO was higher than that of PO. A possible reason for this may be the obvious difference of oxidation degree that appeared between them.

### 2.3. Analysis of Chemical Properties and Fatty Acid Composition

Table 2 shows the chemical parameters of vegetable oil heated for 36 h. Acid value (AV) reflects the content of free fatty acids in oil and increases when oil appears rancid. AV of all samples increased with the heating temperature. Among four oils, the AV of LO was the highest, followed by SO, RO, and PO, and the AV of LO heated at 180 °C for 36 h reached 10.45 mg/g. The change of AV with temperature was obvious, which could be considered one of the main parameters for reflecting the quality and safety of oil. Peroxide value (PV) reflects the content of hydroperoxides in oil, and both AV and PV can reflect the degree of rancidity of oil. PV is an important basic physical and chemical index in the quality evaluation of vegetable oil. PV of four kinds of vegetable oils varied with heating temperature. PV of all oil samples heated at 120 °C was much higher than that of being heated at 150 °C and 180 °C, indicating that hydroperoxides were accumulated and oxidation reaction occurred slowly at this temperature. For oil samples heated at above 150 °C, the decomposition rate of hydroperoxides in the oil was significantly accelerated, and thus the PV declined. The hydroperoxide produced at the initial stage of heating is a reaction intermediate, and its formation rate depends on the availability of oxygen and temperature, which is relatively stable at room temperature [35]. At this time, the carbon chain of fatty acids in vegetable oil is not broken. However, it is easy to decompose into alkoxy groups once hydroperoxide is formed. Then, in the presence of metal or at high temperature, the bonds on both sides of the carbon atom connected with “-OOH” are further broken to form aldehydes, ketones, acids, esters, alcohols, and short chain hydrocarbons. When the content of hydroperoxide reaches a certain level, the decomposition rate would be greater than the formation rate, Thus, the content of hydroperoxide decreased or fluctuated [36]. Therefore, PV is not suitable to characterize the oxidation degree of oil at high temperature. p-Anisidine value (p-AV) reflects the secondary oxidation products of aldehydes of oil oxidation. The increase of aldehyde content in the heated vegetable oil system could be attributed to two aspects including secondary lipid oxidation formed by the degradation of hydroperoxides and thermal oxidation of saturated fatty acid under high temperature [37]. It can be seen from Table 2 that the p-AV in four oils increased with the heating temperature due to the fact that the oxidation rate of unsaturated fat increases at high temperature, and the oxidation of saturated oil molecules produces more aldehydes. Compared with different vegetable oils, the p-AV of LO was the highest and the p-AV of LO heated at 180 °C for 36 h reached 363.47, while p-AV of PO was the lowest, which was consistent with the conclusion that unsaturated esters were more easily oxidized. The change of p-AV with temperature was obvious, which could better characterize the oxidation degree of different vegetable oils during heating.

Table 2 also shows the change of main fatty acid content of four kinds of vegetable oils at different temperatures after being heated for 36 h. On the whole, the content of unsaturated fatty acids in four kinds of vegetable oils decreased with heating temperature. The reason for this may be that during thermal oxidation, a variety of chemical reactions take place in vegetable oil, including the decomposition of triacylglycerol into diacylglycerol, monoacylglycerol, free fatty acid, and glycerol, the formation of unsaponifiable substances by triacylglycerol polymer, and oxidation to produce volatile compounds, such as aldehydes, alcohols, ketones, and hydrocarbons, leading to the decrease of absolute content of saturated fatty acids and unsaturated fatty acids [38]. It can be seen from Table 2 that heating has an effect on different types of fatty acids. The higher the temperature was, the faster the content of fatty acids decreased. The initial fatty acid contents of PO, RO, SO, and LO were 76.58 mg/100 mg, 80.90 mg/100 mg, 74.74 mg/100 mg, and 73.87 mg/100 mg, respectively. The corresponding values of samples heated at 180 °C for 36 h decreased to 66.23 mg/100 mg, 69.80 mg/100 mg, 67.23 mg/100 mg, and 58.81 mg/100 mg, respectively. Obviously, LO was the most unstable among four vegetable oils. This was because the bond dissociation energy of allyl hydrogen is about 10 kca/mol higher than that of diallyl hydrogen [39]. After being heated for 36 h at 180 °C, the contents of palmitic acid (c16:0), oleic acid (C18:1), linoleic acid (C18:2), and α-linolenic acid (C18:3) decreased. During the heating process of the same oil, the degradation rate of unsaturated fatty acids was faster than that of saturated fatty acids, and that of polyunsaturated fatty acids was faster than that of monounsaturated fatty acids. Therefore, for different types of vegetable oils, the higher content of unsaturated fatty acids was accompanied by worse thermal oxidation stability and a greater tendency towards being oxidized.

### 2.4. Analysis of Degradation Products

Volatile compounds in thermally oxidized oil, such as aldehydes, alcohols, acids, ketones, and hydrocarbons, are the break down products of the secondary oxidation products. The main alcohols, aldehydes, and alkanes in the four heated oils are listed in Table 3. Among these, (*E*)-2-decenal, 2-undecenal, and (*E*,*E*)-2,4-decadienal were the major volatile compounds and considered to be the main degraded products, followed by acids, alcohols, and alkanes. The formation mechanism includes the reaction of the radicals with the carbon chains to produce a mixture of conjugated diene hydroperoxides, and the cleavage of hydroperoxides produces the aldehydes mentioned above [40]. The volatiles in the same oil at different temperatures were similar, while those in different oils were significantly different. After the beginning of the chain reactions, 2-undecenal, nonanal, and octanal are produced via the hemolytic cleavages of 8-hydroperoxide, 9-hydroperoxide, and 11-hydroperoxide, respectively [36]. The volatile components, such as hexanal, 2-pentenal, (*E*,*E*)-2,4-heptadienal, and (*E*,*E*)-2,4-decadienal, in LO mainly resulted from the degradation of linolenic acid. Similar studies describing the formation of 1-pentanal, hexanal, and 2,4-decadienal from linoleic acid have been previously reported in the literature [18].

The main oxidative volatiles were 1-octen-3-ol, (*E*)-2-heptenal, (*E,E*)-2,4-Heptadienal, (*E*)-2-decenal, 2-undecenal, (*E*,*E*)-2,4-decadienal, and nonanal, and these products increased sharply with increased heating temperature. (*E*,*E*)-2,4-decadienal was the main volatile compound (1518.29 mg/kg) in SO when heated at 180 °C, and is formed by the degradation of linoleic acid 11-hydroperoxide between the 10th and 11th carbons. (*E*)-2-decenal generates from the same breaking sites of oleic acid 11-hydroperoxide, while (*E*,*E*)-2,4-heptadienal is the degradation product of linolenic acid 8-hydroperoxide between 7th and 8th carbons. Moreover, the aldehydes in RO or SO were higher than those in PO, revealing that the high amounts of oleic acid and linoleic acid with double bonds were more likely to be oxidized to aldehydes. The alkanes obtained from RO were more complex than those obtained from SO, indicating that the acute homolytic cleavage of C-C linkage occurs during the heating process. Among these volatile hydrocarbons, tetradecane and pentane were the major components.

The volatiles detected in the PO samples contained three unique alkanes, including dodecane, tridecane, and tetradecane, and two long chain aldehydes of undecanal and tridecanal. Another notable variation in the heated PO was that the short chain volatiles such as 1-pentanol, hexanal, and (*E*)-2-heptenal significantly decreased at 150 °C and 180 °C. A similar situation was also observed in the case of LO. The oxidized PO contained the highest content of volatiles among the four vegetable oils, while LO contained the lowest. These significant differences in the components and concentrations were owing to the varying fatty acid profiles of PO and LO. As shown in Table 2, the main fatty acids in PO were palmitic acid and oleic acid, while linolenic acid was the main fatty acid in LO. Typically, the greater the number of double bonds, the easier the occurrence of fatty acid oxidation. Hence, the oxidation rates of the three main fatty acids were as follows: linolenic acid > oleic acid > palmitic acid. After 36 h of heating, the LO samples were completely oxidized. Linolenic acid was rapidly converted into hydroperoxides, and then degraded into volatiles. However, prolonged heating resulted in the continuous formation of long chain polymers and cyclic structures. Therefore, compared with LO, the volatiles detected in PO exhibited more diverse and complicated features. The information obtained from the volatile component profile highlighted the complexity of the degraded products. The main volatile compounds, such as (*E*,*E*)-2,4-decadienal, 2-undecenal, and (*E*)-2-decenal, clearly showed the breakage sites. Based on the structures of the volatile components, the corresponding fatty acid chains were determined, and the degradation behavior was revealed.

**Figure 3:**
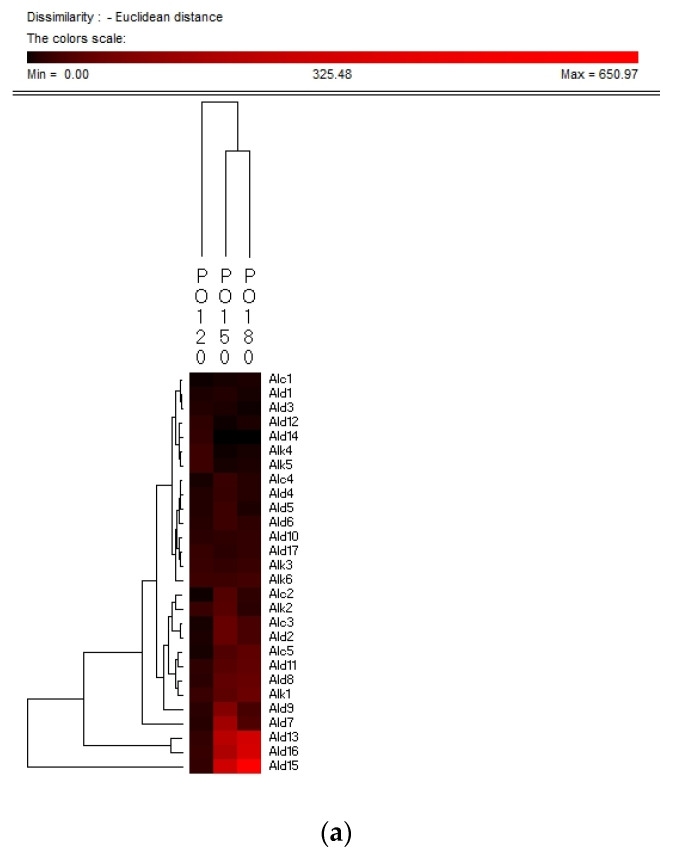

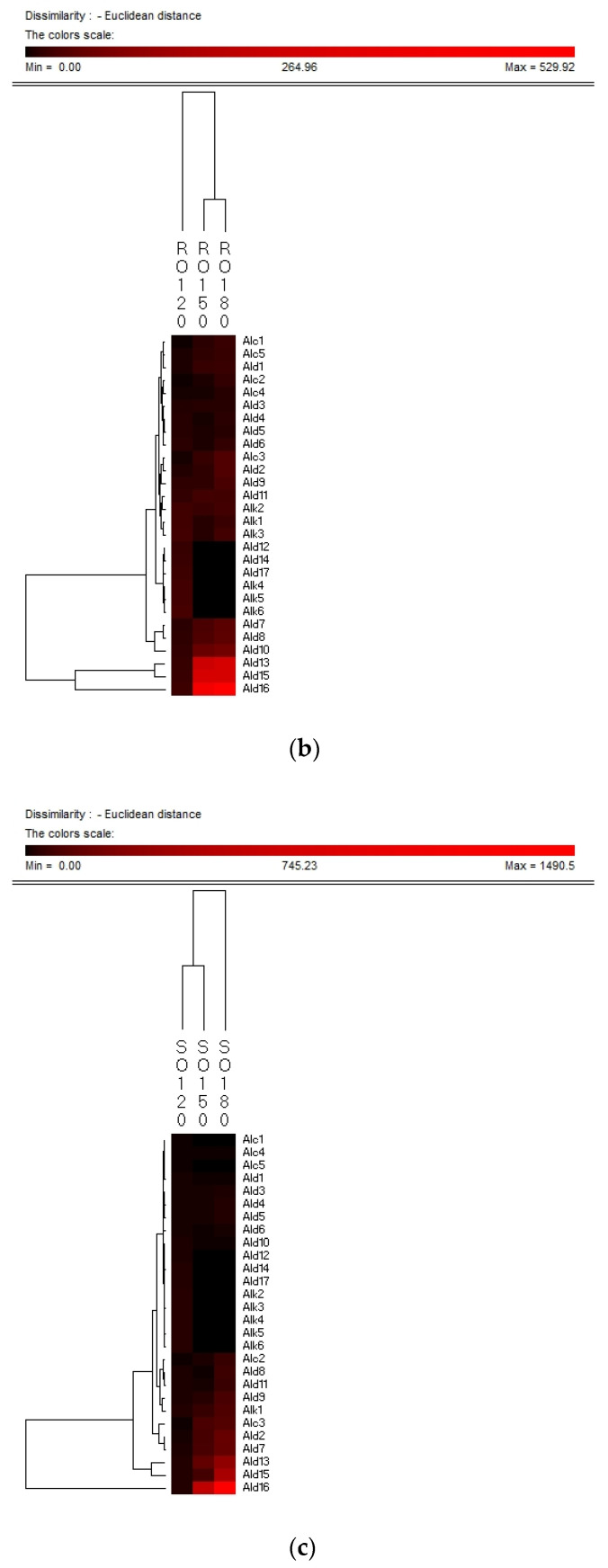

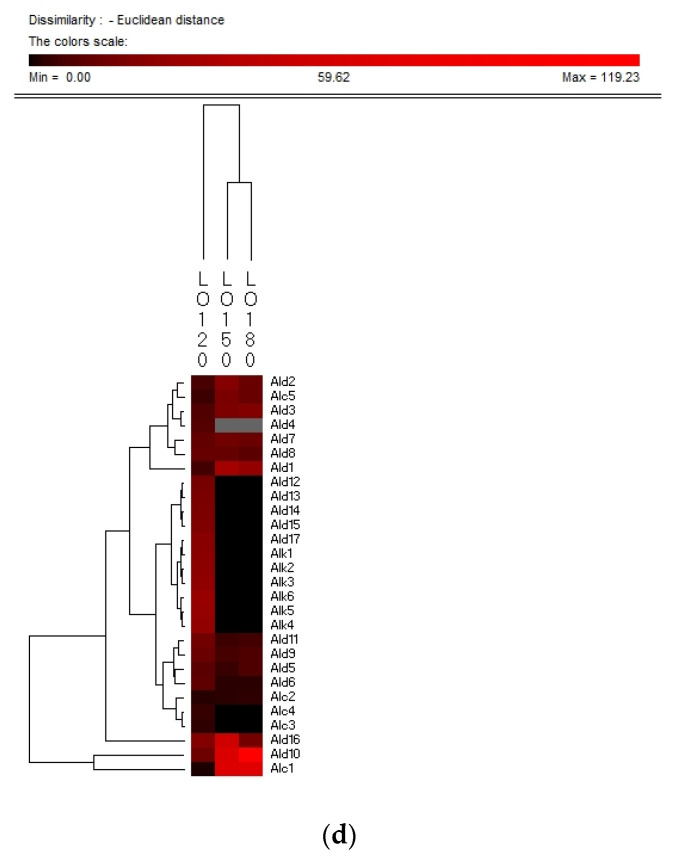
Hierarchical clustering analysis (HCA) chart of GC-MS analysis of volatiles of PO (**a**), RO (**b**), SO (**c**) and LO (**d**).

Hierarchy clustering analysis (HCA) is a basic method for investigating the data in which the natural groupings of samples were characterized by the values of measured features. In present study, HCA was applied for specifying the differences of volatile compounds among oxidized vegetable oils with different heating temperature and selecting the representative indicators for oil oxidation. Figure 3 shows the HCA resulting dendrogram of volatiles during frying of four vegetable oils by characterization of the values of measured volatile data for specifying the differences of volatile profiles among them and signal intensity based on volatile values. As shown in Figure 3, two clusters were identified in four vegetable oils, indicating that there were noteworthy differences among oil samples of different frying temperatures. The first cluster was the PO, RO, and LO samples fried at 120 °C. The frying PO, RO, and LO samples fried at 160 °C and 180 °C were located in the second cluster. While SO samples fried at 120 °C and 150 °C were detected as the first cluster, samples fried at 180 °C were assigned to the second cluster, indicating that SO samples fried at 120 °C and 150 °C possessed lower contents of volatile indicators for oil oxidation than those fried at 180 °C, which could explain why the area of the second cluster was much brighter than that of the first cluster. Besides, the brighter section could be observed at the bottom right corner of the figure. The brighter area corresponded to the much higher contents of volatiles possessed by frying oils. According to Figure 3, PO, RO fried at 150 °C and 180 °C and SO fried at 180 °C contained higher amounts of Ald13 ((*E*)-2-decenal), Ald15 (2-undecenal), and Ald16 ((*E,E*)-2,4-decadienal). However, PO, RO, and SO fried at 120 °C and SO fried at 150 °C contained lower amounts of these typical volatile compounds as the indicators of oil oxidation because the area of this cluster was darker compared with another cluster. The relatively higher contents of Ald13, Ald16, and Ald15 were clustered into one group and these three volatiles increased with the oxidation degree of oil, which indicated that they were representative indicator for lipid oxidation. Regarding LO, the volatiles could be divided into two groups, among which group I contained the samples fried at 120 °C and group II contained samples fried at 150 °C and 180 °C which were rich in Ald16 ((*E*,*E*)-2,4-decadienal), Ald10 ((*E*,*E*)-2,4-heptadienal), and Alc1 (1-penten-3-ol), and the amount of these three volatiles increased with the oxidation degree of LO. The area of the first cluster (LO fried at 120 °C) was much darker, indicating the lower level of flavor components in oxidized LO. Therefore, flavor indicators for oil oxidation of PO, RO, and SO were (*E*)-2-decenal, 2-undecenal, and (*E*,*E*)-2,4-decadienal, and those of LO were (*E*,*E*)-2,4-decadienal, (*E*,*E*)-2,4-heptadienal, and 1-penten-3-ol. The main volatile indicators of oil oxidation in PO, RO, and SO selected by HAC were the same because the high content of oleic and linoleic acid in these three vegetable oils induced the high content of degradation products of oils. Among the three selected oxidation indicator of lipid oxidation, (*E*)-2-decenal and 2-undecenal were produced by homolytic cleavages on the alkoxyl intermediate group of oleate hydroperoxides, while (*E*,*E*)-2, 4-decadienal is reported to be formed by a classical free radical reaction of the decomposition product of oleic and linoleic 9-hydroperoxide [23,41]. As for LO, due to the high proportion of linoleic and linolenic acid, correspondingly high contents of volatile compounds, such as (*E*,*E*)-2,4-heptadienal and 1-penten-3-ol, produced by the oxidation of linolenic acid were formed [42].

### 2.5. Model Establishment and Verification by ANN

**Figure 4:**
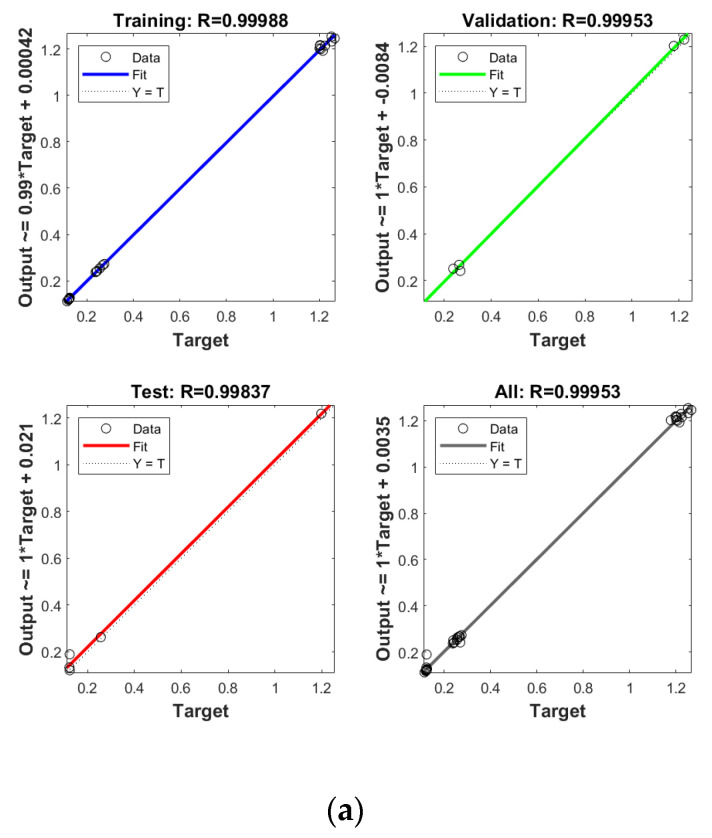

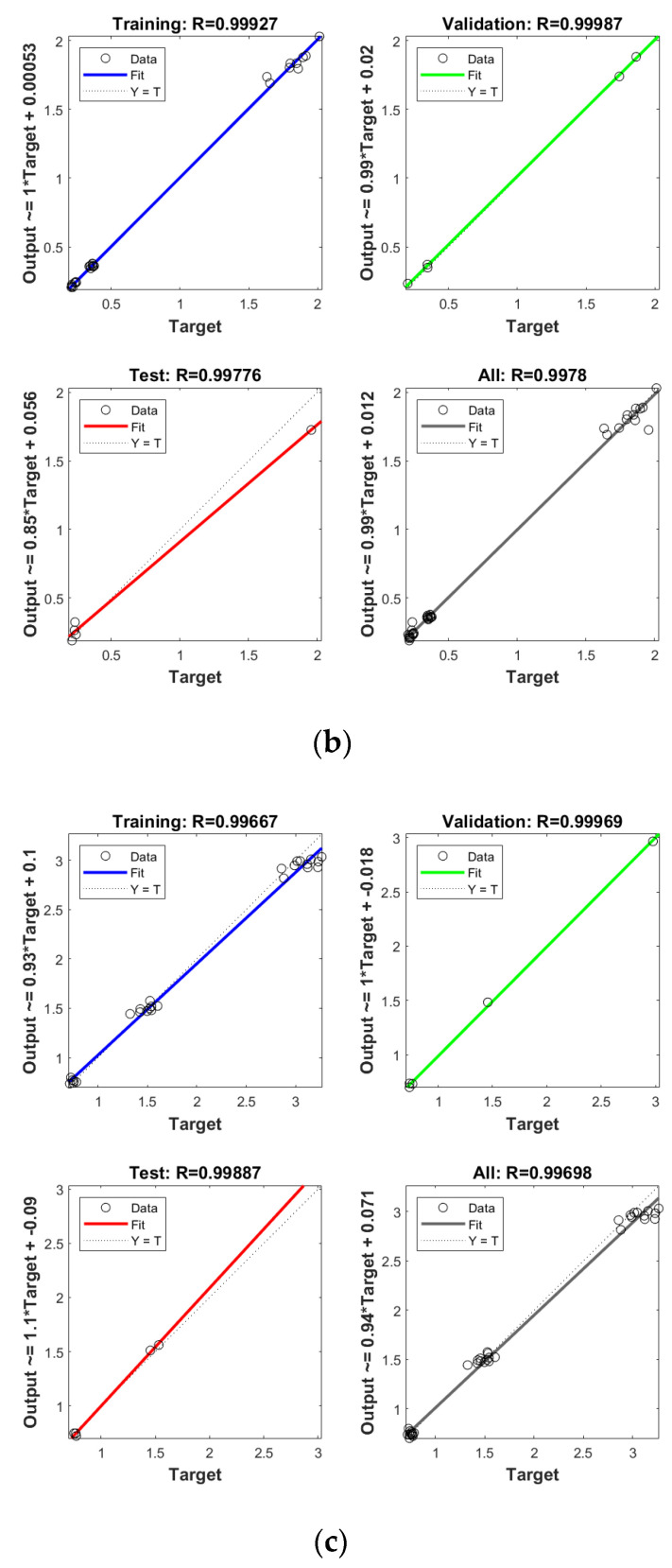

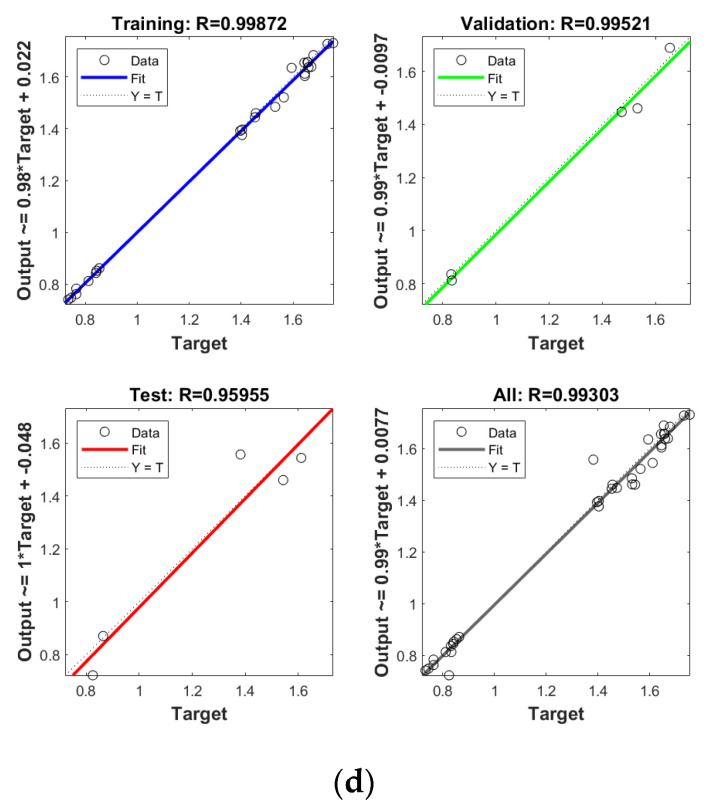
Establishment of prediction model by ANN for free radical of thermal oxidized PO (**a**), RO (**b**), SO (**c**) and LO (**d**).

In order to establish the prediction models for the free radical change of thermal oxidized oils, the chemical data (AV, p-AV), main unsaturated fatty acids (C18:1, C18:2, C18:3), and typical volatile indicators for oil oxidation were selected as the input layer neurons and the free radical level of vegetable oils was used as the output layer neuron. The parameters were randomly divided as training set, validation set, and testing set with the proportion of 70%, 15%, and 15%, respectively. The Levenberg–Marquardt and gradient descent momentum learning function were selected as the training algorithm. The model tuning was achieved based on the number of neurons in the hidden layer. The number of neurons in the hidden layer was adjusted continuously through the test of practical training. When the number of neurons in the hidden layer of ANN was 10, the comprehensive comparison results of R and MSE between the training set and the test set was the best. Moreover, cross-validation on model training was included in the ANN model, analyzed using MATLAB R2018b. ANN has been successfully applied in food to assess aspects of a kinetic model, physicochemical properties, and to support quality analysis and intelligent control design. Deep learning such as ANN has higher recognition accuracy on large sample data sets compared to traditional machine learning [43]. As listed in Figure 4, the predicted values of different oil samples obtained by the ANN method were in high correlation with the measured values, and the R values of the training set were above 0.99. The accuracy and stability of the prediction model was also verified by the results of the high R of validation set with the values of 0.99, 0.99, 0.99, and 0.99 for PO, RO, SO, and LO, respectively, indicating the good prediction ability of these models. Table 4 shows the mean square error (MSE) and coefficient of determination (R2) of the different free radical prediction models for evaluating the performance of fitting and predicting. It can be seen in Table 4 that the R2 values of different free radical models were all above 0.97 (the high R2 corresponding to low MSE) and no significant difference was found between predictive values and true values among methods (*p* > 0.05), and the small predicted and measured values were observed, which indicated that ANN could be used as accurate prediction model for free radical of vegetable oils during thermal oxidation. This is the first time that the ANN was applied to predict the free radicals of vegetable oils. Previous studies on the free radicals in fats and oils were usually determined by EPR, and results showed that the amount of formed free radicals in oxidized fats and oils increased with the increase of oxidized time [34,44], in agreement with our results. The application of ANN in food focuses on the kinetic model, physicochemical properties, quality analysis, and intelligent control design [24]. Sun et al. (2019) used ANN to establish a predictive model for flavor changes in ginger during the drying process, and results showed the model fitted well and a smaller mean square error was observed between the true value and predicted values [27]. The correlation between the free radical and input layer neurons of ANN in oils is shown in Table 5. It should be noted that there was a significant correlation between the free radical amount and input layer neurons of ANN in SO (correlation coefficients were above 0.9). As to LO, the free radical had a high correlation with input layer neurons of ANN except for Ald16 ((*E*,*E*)-2,4-decadienal). The free radical was only highly correlated with Ald15 (2-undecenal) and Ald16((*E*,*E*)-2,4-decadienal) in PO. As shown in Table 4, free radical in RO was relatively correlated with AV, C18:3, and Ald16 ((*E*,*E*)-2,4-decadienal). The same data were analyzed by a linear regression model (LRM), and results showed that the R2 of LRM model applied in PO, RO, SO, and LO was 0.9634, 0.9564, 0.9521, and 0.9462, respectively, which were lower than those of ANN models. The comparison between ANN and LRM indicated that ANN had higher recognition accuracy on data sets compared to the traditional machine learning methods such as LRM. Table 6 shows the values of two factors which were used for quantifying the uncertainty of results. As indicated, the values of p-factor for four ANN models were satisfactory and the predicted lake levels were enclosed by the 95% prediction interval, and the values of d-factor were all less than 1, which indicated that the four ANN models had less uncertainty.

## 3. Materials and Methods

### 3.1. Materials

PO, RO, SO, and LO were donated by Wilmar International Ltd. (Shanghai, China). These raw materials were refined neatly, without the addition of antioxidants. DMPO (99% purity) as a spin trap and 2,4,6-collidine (99% purity) as an internal standard were purchased from J&K Chemical Ltd. (Shanghai, China). DMPO was refined using activated carbon/benzene and then stored in toluene (2 M) at –80 °C (filled with nitrogen) until use. Fatty acid methyl ester standards (37-component bulk mix) and fatty acid (C21:0) methyl ester were obtained from Sigma-Aldrich Corporation (St. Louis, MO, USA).

### 3.2. Preparation of Purified Oils

The residual minor compounds in the vegetable oils were removed by silica column purification. A mixture of silica gel/clay/activated carbon/diatomite (12:6:2:1 *m*/*m*) was added to the column (100 × 8 cm). The oils were extracted with an equal volume of n-hexane at a constant flow rate by the negative pressure generated by the peristaltic pump from the top of the column. The residual n-hexane in the effluents was removed by rotary evaporation and drying with nitrogen. The purified oils were stored at –20 °C until use.

### 3.3. Thermal Oxidation of Oils

The oil samples (400 mL) were individually heated in an oil bath (DF-101S, Jinnan Instrument Manufacturing Co., Ltd., Jinnan, China) for 36 h at 120 °C, 150 °C, and 180 °C. The temperature was periodically tested using a calibrated thermometer. Throughout the heating process, no fresh oil was replenished, and the oil bath was stirred with a magnetic rotor in the dark. The thermally treated oil (20 mL) was ladled out at time intervals of 4 h for analysis. The oil samples were prepared in triplicate and refrigerated until analysis.

### 3.4. Detection of Fatty Acid Composition

The fatty acid profile was analyzed using a gas chromatography system (Shimadzu GC-2010, Shimadzu Corporation, Kyoto, Japan), equipped with a flame ionization detector and a column (60 mm × 0.32 mm × 2.5 μm, TR-FAME 260M154P, Thermo Fisher Scientific, Waltham, MA, USA). An internal standard, fatty acid (C21:0) methyl ester, was dissolved in n-hexane (5 mg/mL) and 100 μL of this solution was added into the weighed oil. The temperature of the injection port was maintained at 250 °C. The program used was as follows: 60 °C for 4 min, 60–170 °C at a heating rate of 5 °C/min, 170 °C for 15 min, 170–220 °C at 2 °C/min, and 220 °C for 15 min. High purity nitrogen at a rate of 20 mL/min was used as a carrier gas. The individual fatty acids were identified by comparison with the retention times of the constituents of the 37-component fatty acid methyl ester standard mixture. Each fatty acid peak was quantified using the internal standard. The experiments were performed in triplicate.

### 3.5. Analysis of the Chemical Properties and Fatty Acid Composition of Oils

Acid value (AV), peroxide value (PV), and p-anisidine value (p-AV) of the oil samples (in Section 2.2) were measured according to the AOCS method Cd 3d-63, Cd 8-53, and Cd 18-90, respectively [45].

### 3.6. Oils Analysis by EPR

The EPR signals of the spin adducts were detected at 120 °C using a Bruker EMXplus-10/12 spectrometer (Bruker Corporation, Billerica, MA, Germany) at 9.85 GHz, equipped with a temperature control unit (Bruker ER 4141 VT-I). The experiment was performed following the procedure described by Chen et al. with slight modifications [23]. A portion of the sample (100 µL) degassed by nitrogen was mixed with 200 µL of 100 mM DMPO in a quartz tube with a diameter of 4 mm, and the tube was inserted into the resonant cavity. The EPR signal intensities of the mixtures were recorded to analyze the fluctuations of the signal intensities. Thereafter, the EPR spectra of the heated oils were recorded immediately after stabilization under dark every 8 h. The parameters were set as follows: center field: 3354 G; sweep width: 100 G; sweep time: 60 s; resolution: 1024 points; microwave power: 20 mW; modulation amplitude: 1.0 G; conversion time: 1.28 ms; modulation frequency: 100 kHz; time constant: 20.48 ms. The radical spin adduct content was determined instantly after the heated oil was ladled out from the beaker at definite intervals. The experiments were performed in triplicate to prevent the difference between free radical distribution and avoid hindering the continuation of the degradation process.

The identification of radical species was carried out by computer simulation of the experimental EPR spectrum based on the calculation of g value and hyperfine coupling constants. Hyperfine splitting constants of experimental spectra were calculated by Bruker Xenon software 4.2 after optimizing signal-to-noise ratios.

The calculation of the number of spins generated in samples was obtained by Bruker Xenon software based on the correction of Baseline Correction and the application of quadratic integral.

### 3.7. Volatile Compounds Analysis by Head Space Solid Phase Microextraction GC-MS

The automatic headspace solid-phase microextraction fibers coated with 50/30 μm divinylbenzene/carboxen/polydimethylsiloxane (DVB/CAR/PDMS) were inserted into the top of a 20 mL headspace vial and retained for 30 min. Each vial contained 0.8 g of the heated oil samples (36 h) and the stirring temperature was set at 50 °C. In addition, 5 μL of 2,4,6-collidine (2.0095 mg/mL in hexane) was added to the samples as an internal standard. Before the analysis, the fiber was desorbed in the injector port of the GC system for 30 min at 250 °C under a stream of helium. After the head space extraction, the desorption was again carried out in the injector port at 250 °C for 5 min without split.

The volatile compounds induced from thermally oxidized oils were detected using a GC-MS/MS instrument (TSQ Quantum XLS, Thermo Fisher Scientific, Waltham, MA, USA) employing a DB-Wax column (30 m × 0.25 mm × 0.25 μm, J&W Scientific, Folsom, CA, USA). The program for the column temperature was set as follows: initial hold at 45 °C for 2 min, 45–180 °C at 3 °C/min, 180–240 °C at 10 °C/min, and 240 °C for 7 min. Further operating parameters of the MS included the electron impact mode (70 eV), ion source temperature of 240 °C, quadrupole filter temperature of 150 °C, and MS range of 33–400 amu. The identification of these compounds was performed by matching the data with the reference mass spectra available in the MS libraries (NIST 14 & WILEY 8.0). The experiments were performed in triplicate.

### 3.8. Uncertainty Measurement of ANN Model

In order to measure and compare the uncertainty related to the results of ANN and ANFIS models, some objective criteria are needed. In present study, we used d-factor and p-factor to evaluate the uncertainty related to the results of ANN models based on the method of Abbaspour et al. [46].

### 3.9. Statistical Analysis

The results were presented as mean values ± standard deviation (SD). Significant differences among samples were assessed statistically by one-way analysis of variance (ANOVA) with Duncan’s t-test using the SPSS (Version 19.0, SPSS Inc., Chicago, IL, USA) at a significance level of 5%. HCA was conducted using PermutMatrix (Version 1.9.3) by dissimilarity analysis of Euclidean distance based on McQuitty’s method. The ANN model was established using MATLAB R2018b (MathWorks, Natick, MA, USA).

## 4. Conclusions

The thermal process accelerated lipid free radical reaction in four vegetable oils. Lipid free radical reactions were more prone to be triggered in LO and SO than PO and RO and further resulted in different initial increase rates of EPR signal intensity. The initiation and propagation stages of the lipid free radicals depended largely on the unsaturation of the main fatty acid in four oils. The thermal process caused a significant increase of EPR signals. Meanwhile, the high temperature limited the excessive increase of lipid free radicals to a certain extent since the high concentrations of free radicals were unstable and highly reactive at high temperature. Consequently, the thermal process accelerated the radical chain reaction and promoted the formation of volatile compounds. GC-MS results showed that (*E*)-2-decenal, (*E,E*)-2,4-decadienal, and 2-undecenal were the main volatile compounds in the four oxidized oils. These unsaturated aldehydes were the degradation products from 8- and 11-hydroperoxides of oleic acid, linoleic acid, and linolenic acid. Meanwhile, three long-chain alkanes, dodecane, tridecane, and tetradecane, were found in PO, which were formed by the degradation of palmitic acid and stearic acid reacted with free radicals at random sites. Besides, the oxidized PO and LO contained the highest and lowest contents of volatiles, respectively.

HCA analysis illustrated that (*E*)-2-decenal, 2-undecenal, and (*E,E*)-2,4-decadienal were the main indicators for the thermal oxidation of PO, RO, and SO, while (*E,E*)-2,4-decadienal, (*E,E*)-2,4-heptadienal, and 1-penten-3-ol were the potential markers for the thermal oxidation of LO. Quantitative prediction models of free radicals in vegetable oils during thermal oxidation were established by ANN based on the variables of oil properties. The high coefficients R2 of free radical values obtained by models (more than 0.97) support the excellent prediction accuracy of ANN. In general, the R2 and MSE of the calibration and prediction sets as well as the small difference between the true and predicted free radicals indicated that the established ANN models based on traditional chemical parameters and volatile profiles could be used to monitor the free radical content of thermal oxidized vegetable oil.

## Figures and Tables

**Table 1:** OSI of four kinds of vegetable oils.

Oil	PO	RO	SO	LO
OSI (h)	13.06	8.85	2.42	0.75

**Table 2:** AV, PV, p-AV and main fatty acid composition of four vegetable oils heated for 36 h.

Oil Type	Heating Temperature (°C)	AV (mg/g)	PV (mmol/kg)	p-AV	Main Fatty Acids (mg/100 mg)				
					C16:0	C18:0	C18:1	C18:2	C18:3(n−3)
PO	Unheated	0.33 ± 0.03	0.50 ± 0.18	3.15 ± 0.12	29.29 ± 0.68	3.59 ± 0.12	33.56 ± 1.02	8.12 ± 0.11	0.11 ± 0.001
	120	3.36 ± 0.15	53.28 ± 2.36	54.84 ± 2.11	24.71 ± 0.07	3.22 ± 0.10	30.15 ± 0.03	6.42 ± 0.00	0.09 ± 0.001
	150	4.89 ± 0.13	5.13 ± 0.02	76.26 ± 1.81	26.33 ± 0.06	3.20 ± 0.03	29.92 ± 0.06	6.02 ± 0.01	0.08 ± 0.001
	180	7.21 ± 0.13	5.87 ± 0.22	126.14 ± 1.01	27.02 ± 0.04	3.27 ± 0.03	28.75 ± 0.08	5.29 ± 0.03	0.04 ± 0.001
RO	Unheated	0.26 ± 0.01	0.75 ± 0.05	4.07 ± 0.16	2.69 ± 0.01	1.51 ± 0.01	46.29 ± 1.21	16.22 ± 0.41	5.15 ± 0.62
	120	4.21 ± 0.23	62.86 ± 0.12	109.16 ± 3.51	2.72 ± 0.09	1.32 ± 0.01	40.82 ± 0.10	13.31 ± 0.02	4.89 ± 0.34
	150	5.98 ± 0.18	3.36 ± 0.06	125.70 ± 3.51	2.57 ± 0.12	1.45 ± 0.10	40.74 ± 0.08	13.01 ± 0.08	4.26 ± 0.41
	180	8.35 ± 0.25	2.54 ± 0.01	230.70 ± 3.84	2.71 ± 0.14	1.46 ± 0.06	41.99 ± 0.11	12.10 ± 0.02	3.32 ± 0.33
SO	Unheated	0.22 ± 0.00	2.03 ± 0.01	5.61 ± 0.28	3.89 ± 0.02	3.25 ± 0.02	13.62 ± 0.34	51.87 ± 1.52	0.22 ± 0.03
	120	4.56 ± 0.15	59.14 ± 0.16	101.37 ± 1.98	3.87 ± 0.11	3.18 ± 0.03	13.25 ± 0.01	49.14 ± 0.05	0.09 ± 0.001
	150	6.56 ± 0.19	6.45 ± 0.01	153.60 ± 10.73	3.89 ± 0.04	3.30 ± 0.05	13.08 ± 0.01	47.77 ± 0.03	0.06 ± 0.001
	180	8.79 ± 0.20	2.69 ± 0.15	207.26 ± 2.07	3.76 ± 0.01	3.26 ± 0.01	12.75 ± 0.01	45.53 ± 0.03	0.05 ± 0.001
LO	Unheated	0.27 ± 0.02	2.78 ± 0.03	3.65 ± 0.01	3.12 ± 0.01	2.76 ± 0.02	13.15 ± 0.28	11.96 ± 0.38	41.02 ± 1.18
	120	5.75 ± 0.16	20.62 ± 0.05	109.16 ± 9.67	2.97 ± 0.03	2.61 ± 0.01	12.53 ± 0.02	11.24 ± 0.01	37.85 ± 0.05
	150	8.42 ± 0.21	5.23 ± 0.14	211.90 ± 0.07	3.03 ± 0.03	2.55 ± 0.01	11.93 ± 0.01	10.66 ± 0.01	33.33 ± 0.08
	180	10.45 ± 0.20	6.70 ± 0.04	363.47 ± 11.18	3.08 ± 0.09	2.51 ± 0.05	11.49 ± 0.09	10.03 ± 0.02	28.79 ± 0.11

**Table 3:** Volatile compounds of four vegetable oils heated for 36 h.

N	Compounds (mg/kg)	PO			RO		
		120 °C	150 °C	180 °C	120 °C	150 °C	180 °C
Alc1	1-Penten-3-ol	4.13 ± 0.28	5.23 ± 0.38	6.00 ± 0.48	10.70 ± 1.01	19.11 ± 1.08	27.32 ± 1.56
Alc2	1-Pentanol	47.92 ± 3.84	15.58 ± 1.24	9.94 ± 0.83	4.93 ± 0.38	14.59 ± 1.02	28.52 ± 1.84
Alc3	1-Octen-3-ol	68.99 ± 5.70	37.74 ± 3.25	42.94 ± 3.38	18.23 ± 1.06	36.64 ± 3.12	65.32 ± 5.32
Alc4	1-Heptanol	23.68 ± 1.98	11.43 ± 1.01	10.57 ± 1.02	2.68 ± 0.15	8.59 ± 0.38	11.97 ± 1.01
Alc5	1-Octanol	44.14 ± 4.02	59.30 ± 5.08	39.58 ± 3.06	13.84 ± 1.03	17.71 ± 1.24	23.45 ± 1.98
Ald1	(*E*)-2-Butenal	7.98 ± 0.81	3.07 ± 0.18	5.09 ± 0.34	17.28 ± 1.56	18.74 ± 1.66	20.81 ± 1.78
Ald2	Hexanal	69.69 ± 5.70	36.50 ± 3.45	36.68 ± 3.28	14.40 ± 1.03	36.46 ± 3.17	38.98 ± 3.76
Ald3	(*E*)-2-Pentenal	5.12 ± 0.43	1.96 ± 0.13	1.93 ± 0.12	9.78 ± 0.65	10.38 ± 0.93	14.32 ± 0.98
Ald4	Heptanal	21.26 ± 1.58	11.67 ± 1.42	12.99 ± 1.14	3.73 ± 0.27	11.97 ± 1.08	25.40 ± 2.30
Ald5	(*E*)-2-Hexenal	26.87 ± 2.35	7.33 ± 0.62	5.56 ± 0.38	5.44 ± 0.39	8.93 ± 0.74	10.52 ± 0.88
Ald6	Octanal	26.91 ± 2.36	15.60 ± 1.32	16.73 ± 1.48	4.91 ± 0.39	15.61 ± 1.38	17.48 ± 1.53
Ald7	(*E*)-2-Heptenal	171.83 ± 15.22	42.00 ± 4.08	38.86 ± 3.52	31.33 ± 3.06	44.89 ± 4.28	67.51 ± 6.07
Ald8	Nonanal	63.48 ± 5.34	69.06 ± 5.39	89.04 ± 7.36	34.80 ± 2.72	46.02 ± 4.30	61.66 ± 5.37
Ald9	(*E*)-2-Octenal	110.04 ± 9.47	34.31 ± 3.04	55.90 ± 4.28	13.40 ± 1.22	29.31 ± 2.46	34.90 ± 3.05
Ald10	(*E*,*E*)-2,4-Heptadienal	17.82 ± 1.45	19.00 ± 1.92	34.22 ± 3.02	59.47 ± 5.27	70.24 ± 6.83	86.73 ± 8.26
Ald11	(*E*)-2-Nonenal	51.22 ± 5.32	62.36 ± 5.87	78.69 ± 7.45	25.16 ± 1.98	23.89 ± 1.87	40.16 ± 3.45
Ald12	Undecanal	2.39 ± 0.13	7.53 ± 0.68	28.54 ± 2.24	ND	ND	ND
Ald13	(*E*)-2-Decenal	233.11 ± 20.42	314.38 ± 26.53	486.37 ± 35.45	237.38 ± 19.52	267.66 ± 21.67	360.42 ± 31.48
Ald14	Dodecanal	ND	ND	14.48 ± 1.38	ND	ND	ND
Ald15	2-Undecenal	299.72 ± 24.64	650.97 ± 54.73	568.85 ± 52.14	273.41 ± 20.57	264.31 ± 20.38	434.26 ± 38.42
Ald16	(*E*,*E*)-2,4-Decadienal	200.64 ± 16.53	331.49 ± 28.32	424.10 ± 38.44	414.68 ± 36.56	529.92 ± 45.33	684.06 ± 49.85
Ald17	Tridecanal	14.12 ± 1.24	20.12 ± 1.92	30.06 ± 2.88	ND	ND	ND
Alk1	Pentane	57.02 ± 4.95	76.09 ± 7.62	98.66 ± 7.85	10.02 ± 0.93	19.76 ± 1.15	43.68 ± 3.82
Alk2	Heptane	52.28 ± 4.16	14.85 ± 1.05	23.37 ± 1.82	20.53 ± 1.93	25.60 ± 2.04	28.72 ± 1.52
Alk3	Octane	18.66 ± 1.56	23.13 ± 1.96	29.80 ± 2.15	10.31 ± 0.92	26.32 ± 2.03	32.08 ± 2.17
Alk4	Dodecane	1.64 ± 0.10	2.80 ± 0.12	4.84 ± 0.24	ND	ND	ND
Alk5	Tridecane	3.25 ± 0.19	6.49 ± 0.43	8.74 ± 0.75	ND	ND	ND
Alk6	Tetradecane	25.62 ± 2.56	32.63 ± 2.89	45.42 ± 3.40	ND	ND	ND
**N**	**Compounds (mg/kg)**	**SO**			**LO**		
		**120 °C**	**150 °C**	**180 °C**	**120 °C**	**150 °C**	**180 °C**
Alc1	1-Penten-3-ol	ND	ND	ND	66.68 ± 5.63	69.64 ± 5.88	74.93 ± 5.90
Alc2	1-Pentanol	12.81 ± 1.08	49.70 ± 4.74	76.42 ± 6.58	2.44 ± 0.18	3.13 ± 0.15	3.36 ± 0.23
Alc3	1-Octen-3-ol	88.26 ± 6.85	106.21 ± 9.68	173.40 ± 15.08	ND	ND	ND
Alc4	1-Heptanol	0.66 ± 0.08	5.00 ± 0.42	11.23 ± 1.01	ND	ND	ND
Alc5	1-Octanol	ND	ND	ND	18.08 ± 1.28	13.25 ± 1.23	15.25 ± 1.26
Ald1	(*E*)-2-Butenal	0.43 ± 0.03	2.00 ± 0.18	8.72 ± 0.57	32.96 ± 3.02	26.28 ± 1.96	34.52 ± 2.78
Ald2	Hexanal	78.54 ± 5.94	160.77 ± 15.74	180.78 ± 16.70	21.44 ± 1.98	13.49 ± 1.75	12.14 ± 1.10
Ald3	(*E*)-2-Pentenal	11.07 ± 0.94	16.88 ± 1.42	21.47 ± 1.93	18.94 ± 1.46	20.70 ± 2.01	22.98 ± 2.14
Ald4	Heptanal	6.00 ± 0.48	17.94 ± 1.58	24.50 ± 1.95	ND	ND	ND
Ald5	(*E*)-2-Hexenal	11.00 ± 0.89	23.06 ± 2.14	45.67 ± 4.33	4.33 ± 0.32	7.58 ± 0.60	9.55 ± 0.76
Ald6	Octanal	1.67 ± 0.17	11.38 ± 1.05	15.32 ± 1.30	2.62 ± 0.22	2.72 ± 0.34	2.70 ± 0.29
Ald7	(*E*)-2-Heptenal	82.21 ± 7.33	155.68 ± 12.75	186.42 ± 17.08	15.43 ± 1.24	13.66 ± 1.09	12.03 ± 1.03
Ald8	Nonanal	3.38 ± 0.22	63.69 ± 5.36	75.62 ± 6.32	12.16 ± 1.02	10.03 ± 0.85	11.90 ± 1.02
Ald9	(*E*)-2-Octenal	26.33 ± 2.92	85.46 ± 7.66	89.40 ± 7.89	6.48 ± 0.42	7.77 ± 0.62	8.02 ± 0.62
Ald10	(*E*,*E*)-2,4-Heptadienal	1.06 ± 0.12	5.00 ± 0.47	12.64 ± 1.65	64.86 ± 6.22	119.23 ± 10.54	134.72 ± 10.22
Ald11	(*E*)-2-Nonenal	7.47 ± 0.64	57.72 ± 5.46	72.14 ± 6.83	5.11 ± 0.41	5.75 ± 0.45	15.80 ± 1.41
Ald12	Undecanal	ND	ND	ND	ND	ND	ND
Ald13	(*E*)-2-Decenal	147.68 ± 12.05	320.37 ± 26.53	479.60 ± 43.88	ND	ND	ND
Ald14	Dodecanal	ND	ND	ND	ND	ND	ND
Ald15	2-Undecenal	74.86 ± 6.62	432.10 ± 39.77	626.70 ± 53.59	ND	ND	ND
Ald16	(*E*,*E*)-2,4-Decadienal	579.06 ± 48.74	1490.46 ± 78.56	1518.29 ± 79.82	54.72 ± 4.37	16.81 ± 1.12	44.63 ± 4.05
Ald17	Tridecanal	ND	ND	ND	ND	ND	ND
Alk1	Pentane	49.62 ± 4.19	97.63 ± 7.62	157.69 ± 10.18	ND	ND	ND
Alk2	Heptane	ND	ND	ND	ND	ND	ND
Alk3	Octane	ND	ND	ND	ND	ND	ND
Alk4	Dodecane	ND	ND	ND	ND	ND	ND
Alk5	Tridecane	ND	ND	ND	ND	ND	ND
Alk6	Tetradecane	ND	ND	ND	ND	ND	ND

ND: not detected.

**Table 4:** Prediction performance of different models for free radicals in different vegetable oils.

Model	R2	MSE (10^−2^)	True Value	Predicted Value
PO	0.9995	0.0306	1.266	1.2461
			1.225	1.2149
			1.203	1.2142
			1.215	1.1927
RO	0.9978	0.0360	1.802	1.8341
			1.742	1.7394
			1.654	1.6912
			1.957	1.7256
SO	0.9970	0.0599	3.154	3.0071
			3.228	2.984
			3.045	2.9897
			2.987	2.9459
LO	0.9799	0.1487	1.654	1.6884
			1.678	1.6835
			1.659	1.6369
			1.754	1.7299

**Table 5:** Pearson correlation coefficient between free radical and input layer neurons of ANN in oils.

	AV	p-AV	C18:1	C18:2	C18:3	Ald13	Ald15	Ald16
PO	−0.731	−0.653	0.538	0.697	0.568	−0.67	−0.993	−0.86
RO	−0.776	−0.543	−0.381	0.636	0.757	−0.634	−0.39	−0.776
SO	−0.972	−0.997	0.925	0.941	0.997	−0.983	−0.999	−0.958
	AV	p-AV	C18:1	C18:2	C18:3	Alcl	Ald10	Ald16
LO	−0.924	−0.979	0.92	0.958	0.951	−0.988	−0.811	−0.055

**Table 6:** Uncertainty measuring parameters for different ANN models.

	PO	RO	SO	LO
d-factor	0.45	0.52	0.36	0.54
p-factor	0.95	0.96	0.96	0.98

## Data Availability

Not applicable.

## Sample Availability

Samples of the compounds are not available.
